# Supplementary material for: Analytical sensitivity and clinical performance of "COVID-19 RT-PCR Real TM FAST (CY5) (ATGen, Uruguay) and "ECUGEN SARS-CoV-2 RT-qPCR" (UDLA-STARNEWCORP, Ecuador)": High quality-low cost local SARS-CoV-2 tests for South America
Source: PLoS Negl Trop Dis. 2022 Apr 13;16(4):e0010082. doi: 10.1371/journal.pntd.0010082 (PMC9037941; doi:10.1371/journal.pntd.0010082)
Supplement: Attachment — Submitted filename: responseletterECUGENPNTD.docx [file pntd.0010082.s002.docx]

Dear Editor

Please find attached the revised version of our manuscript "Analytical sensitivity and clinical performance of "COVID-19 RT-PCR Real TM FAST (CY5) (ATGen, Uruguay) and "ECUGEN SARS-CoV-2 RT-qPCR" (UDLA-Starnewcorp, Ecuador)": high quality-low cost local SARS-CoV-2 tests for South America".

We believe we have properly address all the reviewers' comments as you will find below on this letter. Our answers are highlighted in red for and easy review process.

Also, the changes made to the manuscript are highlighted in red in the "tracked changes version".

**We recall that we are requesting this manuscript to be considered within the ongoing call for papers "Diagnostic tests in low-resource settings", where PNTD is included.**

We hope you consider now this study suitable for publication at your prestigious journal.

Sincerely

Miguel Angel García Bereguiain, PhD.

One Health Research Group.

Universidad de Las Américas.

magbereguiain@gmail.com

+593996484127

***Reviewer's Responses to Questions***

***1. Methods.***

*Reviewer #1.*

*I have problems with the limit of detection (LOD) calculations made by the authors. It appears that no replicates of the samples were tested to determine the LOD. I would expect a minimum of 4 (but ideally more) for a statistically robust analysis.*

This is a great point. For the ECUGEN kit, the LoD based on 5 replicates is already described on reference 16 (doi: 10.1016/j.ijid.2020.10.047) were the triplex assay in which ECUGEN kit is based is described.

Unfortunately, we did not have access to many reactions for the ATGen kit, that it was complicated to get it from Uruguay by the time this study was done as it was not commercially available in Ecuador. For that reason, we even tried a final reaction volume reduction, and even so, less samples were tested for this kit. So, we could not expend reactions on replicates for the LoD. For that reason, we do not calculate the LoD but estimate it, based on the viral loads of the clinical samples used.

So far, we suggested a range for LoD but we did not say that we calculate it.

*More information on the 119 clinical samples is required. When were they collected? From whom? Had they already been tested? How were they stored?*

*We have now included that information required on the methods section. Samples were collected from individuals attending our clinical laboratory at Universidad de Las Américas in Quito during November and December 2020. As it is indicated now, the samples follow the regular diagnostic process in our lab, so the status regarding SARS-CoV-2 infection was known in advance. All samples included in this study were processed with the alternative methods within the same day of our standard method or stored at -80 C if they were processed later. But all the samples were always processed within a 5 days’ period from collection. Moreover, RNase P is included as a quality control of the samples for all the PCR methods tested, so we can certainly assure that no sample degradation happened, and this is why the alternate PCR methods had such a great sensitivity.*

*Why does the number of samples tested per kit differ (i.e. 109 for the ECUGEN and 80 for the ATGen kit)?*

The kit from ATGen company was not available for sale in Ecuador, so we worked with a donation received from the laboratory in Uruguay. Unfortunately, we did not get as many reactions of ATGen kit as we had for the others.

*Page 5: line 87. What do you mean by “reduced reaction volume”? Did you reduce the volume? If so, why?*

We tested a few samples and we could see how no variation on Ct values was obtained with the ATGen kit if we used a smaller reaction volume that was it is indicated on the manual. As we said on the answer above, we did not get many reactions for this kit, so a slight reduction on reaction volume without affecting the kit performance was a strategy to increase the number of samples tested with that kit. We also made this approach for the Thermo Fischer kit, as this kit is extremely expensive in Ecuador and we had a reduced budget for our study. Nevertheless, we assured that no effect on sensitivity was caused by using a smaller final reaction volume.

*Page 5 line 90-91 and 102 to 103. Please rewrite this sentence for clarity. What do you mean by “consistent results with the first test were considered positive”?*

For line 89-91, we have rewritten the sentence for clarity. What we mean if that the replicate of an inconclusive sample yields again a positive amplification no matter if the Ct is over 37, samples is then classified as positive.

For line 102-103, we have rewritten the sentence for clarity. What we mean if that the replicate of an inconclusive yields again a positive amplification no matter if the Ct is over 37, samples is then classified as positive.

*Page 5 line 97. How were the criteria for positivity (i.e. Ct ≤40) calculated? Please provide details.*

The criteria are based on manufacturer's manual indications. All the 3 kits tested in this study are commercially available kits and not in the house protocols, so the positivity criteria are set at the kits instructions.

*Please identify the statistical programme used for analyses.*

We have now included that information on the methods section.

*Reviewer #2.*

The methods used in this study are the methods described by the different kits' manufacturers assessed in this study. Nevertheless, one point was unclear to me: how was the Positive / inconclusive Cycle Threshold set...this seemed a little arbitrary and could change drastically the conclusions (i.e. samples positives and negatives). It would strengthen the study if the authors could explain how they chose Ct 37 for Taq man, Ct 40 for Ecugen and Ct 35 for Tim Fast.

The criteria are based on manufacturer's manual indications, and we clearly state now that on the methods. All the 3 kits tested in this study are commercially available kits and not in the house protocols, so the positivity criteria are set at the kits instructions.

*Moreover, in the case of the Tim Fast kit only 3.75uL of samples was used (instead of 4uL fort the other kits...this also needs explanation).*

*We have detailed that we used a reduced final reaction volume with ATGen kit in order to increase the number of samples that we could test (we did not count with many reactions of this kits that was not available in Ecuador). So, we simple adjust all the reagents volumes proportionally for a final reaction volume of 15 microliters.*

**2. Results.**

*Reviewer #1:*

*No Response.*

*Reviewer #2.*

The results are clearly presented and the "raw" data are in supplemental material. Nevertheless, for the samples where a duplicate experiment was necessary (for exemple samples 59 and 68) to draw a conclusion on the samples it would be nice to show the result of each replicate. When reading that supplemental figure it was not clear why 59 and 68 were called positive whereas 85 and 98 were called negative (Taq Man kit).

Thanks a lot for the corrections:

- Samples 59 and 68 are considered positive. They were inconclusive as only one viral target amplified on the first run, and when they were repeated, the amplification of that target persisted. We have now added the 2 values of Ct for the two runs.

- Samples 85 and 98 are negative. This was a mistake. They gave a Ct value over 40 and we have now changed that for N/A (not amplification) and TaqMan kit set the threshold at 40.

**3. Conclusions**

*Reviewer #1.*

No Response.

*Reviewer #2.*

*The conclusions of this manuscript are fully supported by the data presented here. This study is of general interest as it shows that alternative methods for SARS-COV-2 detection are as performant as the CDC approved ones and can be use for general testing. The number of samples (#100 ) is good enough to be able to draw solid conclusions.*

We appreciate such a supportive comment to our study.

**4. Editorial and Data Presentation Modifications.**

*Reviewer #1.*

*The English is sufficient but could be improved.*

We have now proofread the manuscript to improve English quality.

*A brief description of the SARS-CoV-2 genome describing all of the target genes would be helpful.*

Good suggestion; we have now included that info on the introduction when the several options for SARS-CoV-2 detection kits are described.

*Last paragraph can be removed Page 9 line 182-193 as I do not think it is relevant to the study.*

We understand reviewer’s opinion, as that paragraph is not related to the clinical performance of the kit. But we kindly request to keep this paragraph. In the context of South America, it is important to point out that regionally produced kits have a great clinical performance. It is not usual to have those type of reagents locally produced in South America, although is normal if other regions of the world. So, this is why we want to let this idea on our manuscript to endorse tech development in our countries. Moreover, considering that South America has been overflow with commercial kits for SARS-CoV-2 detection of poor quality, than despite lacking of Emergency Use Authorization in the country of production (usually industrialized countries), they have been used in South American countries affecting the reliability of SARS-CoV-2 detection.

Reviewer #2.

No Response.

**5. Summary and General Comments.**

Reviewer #1.

*In their manuscript the authors have evaluated the performance of two commercial diagnostic RT-qPCR kits for the detection of SARS-CoV-2. Evaluation of kits is important but it must be done in a systematic and statistically robust way. Accordingly, this manuscript requires revision.*

We appreciate all the reviewer 1 comments that we believe we have addressed properly. Although there are multiple studies evaluating commercial kits for SARS-CoV-2 detection, we carried out the evaluation of the only locally produced kits available in countries like Ecuador or Uruguay. This is why we considered that our study would fit with the call for papers at PNTD addressing "new diagnostic tools at low income settings". The main limitation of our study is the sample size for the kit from Uruguay, but unfortunately we were not able to get more reactions of that kit.

Reviewer #2.

*This study compares the efficacy of 2 novel commercial kits to the CDC approved one for SARS-Cov-2 detection. This study is straight forward and well performed. It is important for each country to be able to implement wide testing with its own resources to avoid problems of shortage. The data are presented clearly and the conclusions are strongly supported by the results.*

We appreciate reviewer 2 for this kind comments. This was the aim of this manuscript: to show that locally produced kits at South American countries are as good as other expensive imported commercial ones.
