# Supplementary material for: Analytical sensitivity and clinical performance of "COVID-19 RT-PCR Real TM FAST (CY5) (ATGen, Uruguay) and "ECUGEN SARS-CoV-2 RT-qPCR" (UDLA-STARNEWCORP, Ecuador)": High quality-low cost local SARS-CoV-2 tests for South America
Source: PLoS Negl Trop Dis. 2022 Apr 13;16(4):e0010082. doi: 10.1371/journal.pntd.0010082 (PMC9037941; doi:10.1371/journal.pntd.0010082)
Supplement: Attachment — Submitted filename: responseletterECUGENPNTDV2.docx [file pntd.0010082.s003.docx]

Dear Editor

Please find attached the revised version of our manuscript "Analytical sensitivity and clinical performance of "COVID-19 RT-PCR Real TM FAST (CY5) (ATGen, Uruguay) and "ECUGEN SARS-CoV-2 RT-qPCR" (UDLA-STARNEWCORP, Ecuador)": high quality-low cost local SARS-CoV-2 tests for South America".

We believe we have properly address all the reviewers' comments as you will find below on this letter. Our answers are highlighted in red for and easy review process.

Also, the changes made to the manuscript are highlighted in red in the "tracked changes version".

We hope you consider now this study suitable for publication at your prestigious journal.

Sincerely

Miguel Angel García Bereguiain, PhD.

One Health Research Group.

Universidad de Las Américas.

magbereguiain@gmail.com

+593996484127

***Reviewer's Responses to Questions.***

*1. Methods.*

*Reviewer #1: No Response.*

*Reviewer #2: no more comments. The authors addressed my comments from the first round of review.*

*I just found a typo page 2 line 24 "with our".*

**Corrected in the manuscript.**

*Reviewer #3: Straightforward methodology with manufacturer provided protocols. Details have been provided accordingly as previous reviewer's suggestion. The sample limitation seems to be a problem but unsolvable. The authors have addressed this concern in the manuscript.*

**Thanks for the input. Yes, unfortunately we did not have access to more reactions of the ATGen kit.**

*2. Results.*

*Reviewer #1: No Response.*

*Reviewer #2: yes*

*Reviewer #3: The supplementary data is weird. For sample ID 20414, there are Ct values for TaqMan 2019-nCoV Assay Kit v1 but no result indication for this sample. Is this sample tested or not?*

**Thanks for the advice. That was a mistake, the sample is positive but we did not fill the column for the result although the Ct was given. Corrected.**

*The LOD range calculation is a pure deduction from the clinical samples. It is not wrong but the evidence is relatively weak.*

**The reviewer is correct. As we have access to more reactions for the ECUGEN kit, we have now added a new table where the LoD is address with multiple replicates for several viral loads.**

**Also, we have added a new Figure 1 where the viral load distribution for N target for all the positive samples tested with three kits tested is detailed (panel A). Also a linear regresion is pesented in panel B to endorse the high level of agreement of both ECUGEN and ATGen compared to TaqMan kit.**

*3. Conclusions.*

*Reviewer #1: (No Response)*

*Reviewer #2: yes*

*Reviewer #3: The aim of this manuscript fits the Plos NTD in terms of focusing the low income countries and their respective situation of molecular diagnosis of COVID-19. The evaluation of these local produced kits may be of interest to relevant countries which have difficulties in accessing the golden commercial kits. However, the need of molecular diagnosis is necessary to global prevention of COVID-19. Such simple and direct testing may provide alternatives for those countries' public health systems.*

**Many thanks for the feed back.**

4. Summary and General Comments

*Reviewer #1: The authors have satisfactorily answered all of the questions put to them. Unfortunately, it seems that some of the shortcomings of their study highlighted by this reviewer were due to a lack of ATGen kits for a complete and robust evaluation. Even though this is not the fault of the authors, it still does not justify the publication of an incomplete evaluation study. I therefore recommend that the authors revise their manuscript and concentrate on the evaluation of the ECUGEN kit alone.*

***We have substantially improved the evaluation of ECUGEN kit by adding a Table 2 with LoD calculation. Also, a new figure 1 analysis the accuracy of both kits. Although ATGen evaluation is not as robust as ECUGEN, the panel B on figure 1 supports that a great correlation to the gold standard TaqMan kit. Moreover, the limitations of the study are properly described. This is why we prefer to keep both South American kits included in the study.***

*Reviewer #2: (No Response)*

*Reviewer #3: A straightforward and simple diagnostic kit evaluation from respective company. This finding can provide alternatives for low income countries to support their need of COVID-19 detection kits. In order to better commercialize the kits, a larger sampling size is definitely needed for displaying confident kit performance. Yet, this paper can still provide preliminary performance quality for the public section to consider.*

**As we have detailed before, we have improved the manuscript with LoD calculation for ECUGEN and a deeper analysis of ATGen and ECUGEN compared to TaqMan as described in Figure 1. So, although the results may be somehow preliminary, the new version manuscript present a more robust evaluation.**
